# Mitochondria-Targeted Triphenylphosphonium-Hydroxytyrosol Prevents Lipotoxicity-Induced Endothelial Injury by Enhancing Mitochondrial Function and Redox Balance via Promoting FoxO1 and Nrf2 Nuclear Translocation and Suppressing Inflammation via Inhibiting p38/NF-кB Pathway

**DOI:** 10.3390/antiox12010175

**Published:** 2023-01-11

**Authors:** Xuyun Liu, Jing Gao, Yizhen Yan, Eleftheria A. Georgiou, Jing Lou, Mengya Feng, Xing Zhang, Feng Gao, Jiankang Liu, Ioannis K. Kostakis, Lin Zhao

**Affiliations:** 1 Center for Mitochondrial Biology and Medicine, The Key Laboratory of Biomedical Information Engineering of Ministry of Education, School of Life Science and Technology and Frontier Institute of Science and Technology, Xi’an Jiaotong University, Xi’an, 710049, China; 2 Key Laboratory of Ministry of Education, School of Aerospace Medicine, Fourth Military Medical University, Xi’an 710032, China; 3 Faculty of Pharmacy, School of Health Sciences, National and Kapodistrian University of Athens, Panepistimiopolis-Zografou, 15771 Athens, Greece; 4 School of Life Sciences, University of Health and Rehabilitation Sciences, Qingdao 266071, China

**Keywords:** TPP-HT, hyperlipidemia, endothelial injury, mitochondrial dysfunction, FoxO1, Nrf2

## Abstract

Hyperlipidemia results in endothelial dysfunction, which is intimately associated with disturbed mitochondrial homeostasis, and is a real risk factor for cardiovascular diseases (CVDs). Triphenylphosphonium (TPP^+^)-HT, constructed by linking a mitochondrial-targeting moiety TPP^+^ to hydroxytyrosol (HT), enters the cell and accumulates in mitochondria and is thus an important candidate drug for preventing hyperlipidemia-induced endothelial injury. In the present study, we found that TPP-HT has a better anti-inflammatory effect than HT. In vivo, TPP-HT significantly prevented hyperlipidemia-induced adverse changes in the serological lipid panel, as well as endothelial and mitochondrial dysfunction of the thoracic aorta. Similarly, in vitro, TPP-HT exhibited similar protective effects in palmitate (PA)-induced endothelial dysfunction, particularly enhanced expression of the mitochondrial ETC complex II, recovered FoxO1 expression in PA-injured human aorta endothelial cells (HAECs) and promoted FoxO1 nuclear translocation. We further demonstrated that FoxO1 plays a pivotal role in regulating ATP production in the presence of TPP-HT by using the siFoxO1 knockdown technique. Simultaneously, TPP-HT enhanced Nrf2 nuclear translocation, consistent with the in vivo findings of immunofluorescence, and the antioxidant effect of TPP-HT was almost entirely blocked by siNrf2. Concomitantly, TPP-HT’s anti-inflammatory effects in the current study were primarily mediated via the p38 MAPK/NF-κB signaling pathway in addition to the FoxO1 and Nrf2 pathways. In brief, our findings suggest that mitochondria-targeted TPP-HT prevents lipotoxicity induced endothelial dysfunction by enhancing mitochondrial function and redox balance by promoting FoxO1 and Nrf2 nuclear translocation.

## 1. Introduction

Globally, prevalent cases of total CVDs almost doubled from 271 million in 1990 to 523 million in 2019, and the number of deaths from CVDs gradually increased from 12.1 million in 1990 to 18.6 million in 2019, and CVDs remain the principal cause of disease burden in the world [1]. Endothelial dysfunction leads to a shift in the properties of the endothelium toward reduced vasodilation, a proinflammatory state, and prothrombotic properties, resulting in accelerated atherogenesis in the early stages of CVDs [2]. Numerous studies have shown that metabolic stresses, such as hyperglycemia, and especially hyperlipidemia, can result in endothelial dysfunction and vascular complications [3]. Elevated FFAs accelerate vascular endothelial dysfunction, which further causes CVDs [4,5]. However, effective targets of intervention for endothelial dysfunction remain largely undefined.

Mitochondria are the primary energy source and play an important role in coupling intracellular and intercellular metabolic cooperation [6]. Abundant evidence demonstrates that impaired mitochondrial oxidative phosphorylation leads to vascular diseases [7]. Additionally, elevated inflammation and oxidative stress, the key risk factors for endothelial dysfunction, are intimately involved in mitochondrial impairment [8]. Intervention means to robust mitochondrial function also have demonstrated to ameliorate endothelial abnormalities [9]. Mitochondria, therefore, constitute a promising target for the development of novel agents for the intervention in endothelial damage and even CVDs [10]. Thus, we attempted to acquire a newly designed compound to maintain mitochondrial homeostasis by targeting mitochondria and thus preventing hyperlipidemia-related mitochondrial abnormalities and oxidative stress to protect against endothelial injury.

Hydroxytyrosol (HT), a phenolic phytochemical that exists in olive leaves and oil, is a valuable compound that exhibits various health-promoting effects, such as antioxidant, anti-inflammatory and anticancer activity [11,12]. In addition, HT has been demonstrated to protect against endothelial dysfunction [13]. However, HT applications, such as dietary supplements and food stabilizers in nonaqueous media are limited, due to their hydrophilic character [14]. The triphenylphosphonium (TPP^+^) moiety has been extensively used to target molecules into mitochondria. Synthesized TPP^+^ derivatives, such as MitoQ, have been described as promising targeted therapies for vascular damage [15]. To make it easier for HT to cross biological membranes and accumulate in mitochondria at high concentrations, we designed and synthesized TPP-HT with enhanced lipophilic properties, which is linked to TPP^+^ through a covalent bond to the side chain of HT. In the present study, we propose that TPP-HT may have high potential in targeting mitochondria and protecting against endothelial injury. In the current study, the protective effects and possible mechanisms of TPP-HT on hyperlipemia-induced endothelial impairment were investigated using in vitro and in vivo models.

## 2. Materials and Methods

### 2.1. TPP-HT Synthesis

To overcome difficulties such as the number of steps, overall yield, and purification problems, we considered a convergent approach using a protected hydroxytyrosol analog as starting material. Thus, 3,4-dibenzyloxyphenethyl alcohol reacted with 1,6-dibromohexane to provide an intermediate bromide which was easily deprotected by Pd/C, Et3SiH and treated with triphenylphosphine to afford TPP-HT. The overall yield of the synthetic procedure is ~23%,and the final purity of TPP-HT was greater than 97%.

All the above commercial chemicals were purchased from Alfa Aesar. Melting points were determined on a Büchi apparatus and were uncorrected. ^1^H NMR, ^13^C NMR and 2D spectra were recorded on a Bruker Avance III 600, 400 spectrometer and 200 (Bruker GmbH, Germany), using dimethyl sulfoxide (DMSO-*d_6_*), methanol (CD_3_OD) and chloroform (CDCl_3_) as deuterated solvents and were referenced to TMS (*δ* scale). Flash chromatography was performed on Merck silica gel 60 (0.040–0.063 mm). Analytical thin layer chromatography (TLC) separations were carried out on precoated (0.25 mm) Merck KgaA (Darmstadt, Germany) silica gel F-254 plates. HRMS was obtained from an LTQ-Orbitrap Discovery Mass Spectrometer (Thermo Scientific, Brehmen, Germany). Furthermore, we identified and confirmed the purity of the novel compounds using ^1^H NMR and ^13^C NMR spectroscopy.

### 2.2. Animal Experiments

We purchased male C57BL6 mice (6 weeks old) from the Experimental Animal Center of the Fourth Military Medical University, Xi’an, China (20200139). In this study, all animals were housed in cages in a room kept at 22–28 °C, under a 12 h light/dark cycle, with free access to food and water during the entire experimental process. The mice needed to be randomly divided into four groups as follows: control group (*n* = 8), hyperlipidemia group (*n* = 8), low-dose TPP-HT treated group (*n* = 8) (5 mg/kg/day TPP-HT by gavage once a day for 9 days), and high-dose TPP-HT treated group (*n* = 8) (20 mg/kg/day TPP-HT by gavage once a day for 9 days). P407 (poloxamer 407; Sigma-Aldrich, Darmstadt, Germany) (0.5 g/kg) was used to induce a hyperlipidemia model via intraperitoneal injection for exactly 24 h (Day 10) [16]. After the above manipulation, mice were killed by cervical dislocation for dissection of the thoracic aorta [17]. All animal experiments in our study were performed according to national guidelines. The experiments were approved by the Fourth Military Medical University Animal Care and Use Committee, Xi’an, China. During the whole study, we tried our best to minimize the suffering and the number of animals used.

### 2.3. Hematoxylin and Eosin (H&E) Staining and Immunohistochemistry

Mouse thoracic aortas were fixed with a concentration of 4% paraformaldehyde for less than 48 h. Samples were dehydrated and embedded in paraffin, and 5 mm sections were cut with a Leica RM-2162 (Leica; Bensheim, Germany) for hematoxylin and eosin staining and immunohistochemistry for detecting FoxO1 and Nrf2 expression, which was described in a previous study [18].

### 2.4. Transmission Electron Microscopy

The other part of the mouse thoracic aorta was further fixed with 2.5% glutaraldehyde in 0.1 M PBS (pH 7.2–7.4) for 2 h at 4 °C; then, the arteries were washed three times with PBS and postfixed in 1% osmium tetroxide in PBS for 2.5 h. Furthermore, mouse thoracic aortas were dehydrated in a graded ethanol series, infiltrated with propylene oxide and embedded in Epon 812. Toluidine blue-stained sections (1 mm) were detected by light microscopy; moreover, applicable areas were used for slicing thin sections (LKB-V). The above sections were stained with uranyl acetate and lead citrate; then, they were observed by a transmission electron microscope (H-7650; Hitachi, Tokyo, Japan).

### 2.5. Chemicals

Antibodies against Nrf2 and optic atrophy 1 (OPA1) were purchased from Abcam (Cambridge, MA, USA). Antibodies against FoxO1, p-Erk, Erk, p-p38, p38, p-NF-κB, NF-κB, Mitofusin-1 (Mfn1), Mitofusin-2 (Mfn2), α-tubulin and β-actin were purchased from Cell Signaling Technology (Beverly, MA, USA). Antibodies against complex I NDUFS3 (39 kDa), II SDHB (25 kDa), III UQCRC1 (51 kDa), IV MTCO1 (40 kDa) and V ATP5A1 (55 kDa) were purchased from Invitrogen (Carlsbad, CA, USA). Sodium PA (catalog, p9767) and fatty acid-free bovine serum albumin (catalog, 126575) were obtained from EMD Millipore Corp. (Billerica, MA, USA). TPP-HT in animal and cellular experiments was synthesized by Ioannis K. Kostakis. The P38 inhibitor SB203580 (catalog, ab120162) and NF-κB inhibitor QNZ (catalog, ab141588) were obtained from Abcam Inc. (Boston, MA, USA). The ERK inhibitor U0126 (catalog, S1102) was purchased from Selleck Chemicals (Houston, TX, USA). DCF, RNAiMAX transfection reagent and other reagents were purchased from Invitrogen (Carlsbad, CA, USA). Nonesterified fatty acids, triglycerides, total cholesterol, low-density lipoprotein cholesterol, high-density lipoprotein cholesterol and ATP levels were examined with commercial clinical diagnosis kits according to the instructions (Jiancheng Bioengineering Co., Ltd., Nanjing, China). Cell viability was analyzed using a commercial CCK-8 kit according to the manufacturer’s instructions (Bioscience Bioengineering Co., Ltd., Shanghai, China).

### 2.6. Cell Culture and Treatment

The human aorta endothelial cells (HAECs) used in this study were obtained from BioLeaf (Shanghai BioLeaf Biotech Co., Ltd., Shanghai, China). HAECs were plated on a glass-bottom culture plate with Dulbecco’s modified Eagle’s medium (DMEM) and 10% FBS media supplemented with 1% penicillin-streptomycin and 10 μg/mL heparin in a 95% air and 5% CO_2_ incubator. In addition, the steps for preparing the PA stock solution (200 mM) were described in our previous study [19]. HAECs were first treated with or without a range of concentrations of TPP-HT for 24 h, followed by PA (500 μM) treatment for another 24 h. The treatments for HAECs were performed on subconfluent monolayers.

### 2.7. Cell Viability Assay

HAECs were seeded at a density of 4 × 10^4^ per well in 96-well plates for 24 h; then, HAECs were pretreated with TPP-HT (5–50 μM) for 24 h, followed by PA (500 μM) challenge for another 24 h. The viability of the treatments was analyzed using a commercial CCK-8 kit (Bioscience Bioengineering Co., Ltd., Shanghai, China).

### 2.8. Determination of Reactive Oxygen Species (ROS)

The ROS of HAECs was detected using fluorescence of 2′,7′-dichlorofluorescein (DCFH2-DA). Briefly, HAECs were cultured with serum-free medium supplemented with 10 μM DCFH2-DA for 40 min. Then, two methods through microplate fluorometer and microscopy were applied to detect the fluorescence intensity. The steps of these two methods were the same as those in our previously published study [19].

### 2.9. Confocal Microscopy

HAECs were seeded onto poly-L-lysine-coated coverslips for 24 h and then treated with 10 μM TPP-HT for 24 h. First, HAECs were fixed with 4% paraformaldehyde for 30 min and washed three times with PBS. Then, the cells were treated with 0.2% Triton-X-100 for 5 min (Sigma) and washed with PBS three times at room temperature. Then, the cells were blocked with 3% BSA for 1 h at room temperature and incubated with antirabbit FoxO1 and antirabbit Nrf2 antibodies at 4 °C overnight. After washing three times with PBS, HAECs were incubated with Alexa Fluor 555-conjugated antirabbit IgG (Invitrogen, Carlsbad, CA, USA) antibody. The cells were further washed with PBS three times, stained with DAPI (Invitrogen, Carlsbad, CA, USA), and determined with an LSM 800 fluorescence microscope at 40× magnification (Zeiss, Oberkochen, Germany).

### 2.10. Real-Time PCR

The steps of real-time PCR were the same as those in our previously published study [19]. The primers used are listed in Supplementary Table S1.

### 2.11. SiRNA (Small Interfering RNA) Transfection

SiRNAs were purchased from Genepharma Co. (Shanghai, China). The sequences of FoxO1 siRNA were 5′-CCACACAGUGUCAAGACAATT-3′ and 5′-UUGUCUUGACACUGUGUGGTT-3′. Nrf2 siRNA were 5′-CGCUCAGUUACA ACUAGAUTT-3′, 5′-AUCUAGUUGUAACUGAGCGTT-3′. Scrambled siRNA was used as a negative control. Cells were seeded at 1.5 × 10^5^ cells per well in 6-well plates for transfection. When the density of HAECs reached 20–30% confluence, HAECs were further transfected with FoxO1-targeting siRNA, Nrf2-targeting siRNA or nontargeting control siRNA at a concentration of 25 nM for 24 h before the subsequent TPP-HT and PA treatment.

### 2.12. Western Blotting

The steps of Western blotting were the same as those in our previously published study [19].

### 2.13. Statistical Analysis

All data are shown as the mean ± SEM. Statistical analyses were performed using one-way ANOVA and LSD post hoc analysis (SPSS Inc., Chicago, IL, USA). In this study, values of *p* < 0.05 were regarded to be statistically significant.

## 3. Results

### 3.1. TPP-HT Prevents P407-Induced Serum Lipid Profile Abnormalities and Endothelial and Mitochondrial Injury in the Thoracic Aorta

**Figure 1:**
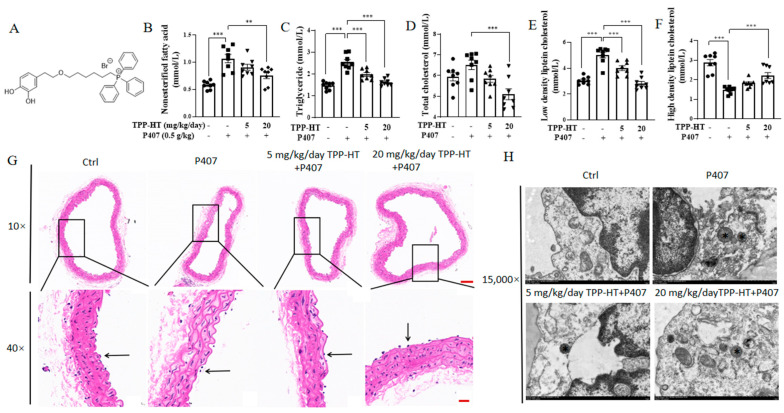
TPP-HT prevents P407-induced serum lipid profile abnormalities and endothelial and mitochondrial injury in the thoracic aorta. (**A**) Chemical structure of TPP-HT. (**B**–**F**) Nonesterified fatty acids, triglycerides, total cholesterol, low-density lipoprotein cholesterol and high-density lipoprotein cholesterol. (**G**) H&E staining of microstructural changes in the thoracic aorta. The arrow indicates thoracic aortic endothelial cells, scale bar = 100 μm (**upper**) and 20 μm (**lower**). (**H**) Ultrastructural changes in the thoracic aorta; asterisk indicates swelling mitochondria, scale bar = 1 μm. Control group (*n* = 8), hyperlipidemia group (*n* = 8), low-dose TPP-HT group (*n* = 8), high-dose TPP-HT group (*n* = 8). Values are the mean ± SEM. ** *p* < 0.01, *** *p* < 0.001.

To investigate the potential benefits of TPP-HT (Figure 1A), our study used an acute hyperlipidemia-induced endothelial injury mouse model. This hyperlipidemia model was constructed by intraperitoneal injection of P407, as described in our previously published work [19]. In a previous study, the concentration of HT in olive oil varied from 1.55 to 14.42 mg/kg [20]. A crude count shows that a Mediterranean diet with 50 g olive oil intake should receive 0.7 mg HT at most per day. A previous study showed that 5 mg/kg/day supplementation with HT for 12 weeks improved white adipose function in high-fat-diet-fed mice [21]. Thus, in the current study, two doses of TPP-HT were chosen (5 and 20 mg/kg/day) to observe the dose effects. TPP-HT was administered by oral gavage at dosages of 5 mg/kg/day and 20 mg/kg/day for 9 days prior to the P407 challenge. Higher nonesterified fatty acid, triglyceride, total cholesterol, and low-density lipoprotein cholesterol levels and lower high-density lipoprotein cholesterol levels in serum were present in the P407 group, which were partially rescued by TPP-HT supplementation at both dosages of 5 and 20 mg/kg/day; apparently, the effect of 20 mg/kg/day was better than the effect of 5 mg/kg/day (Figure 1A–E). To examine whether TPP-HT could ameliorate the structural injury induced by P407, the microstructure of endothelial cells in the thoracic aorta was observed via H&E staining. In comparison with control group, the distance between endothelial cell nuclei and the internal elastic membrane in the P407 group was increased, indicating that swelling of endothelial cells occurred during hyperlipidemia. The abnormal structure was in part reversed in both the low- and high-dose TPP-HT-treated groups (Figure 1F). Moreover, we also examined the effects of TPP-HT on ultrastructural changes in endothelial cells in the thoracic aorta using transmission electron microscopy. As shown in Figure 1G, the hyperlipidemia group showed partially swollen mitochondria compared with the control groups. High-dose TPP-HT supplementation reduced the number of swollen mitochondria, suggesting an improvement in the mitochondrial function of endothelial cells in the thoracic aorta.

### 3.2. TPP-HT Prevents PA-Induced Cellular Lipotoxicity, Increases Inflammation and Decreases eNOS Expression in HAECs

**Figure 2:**
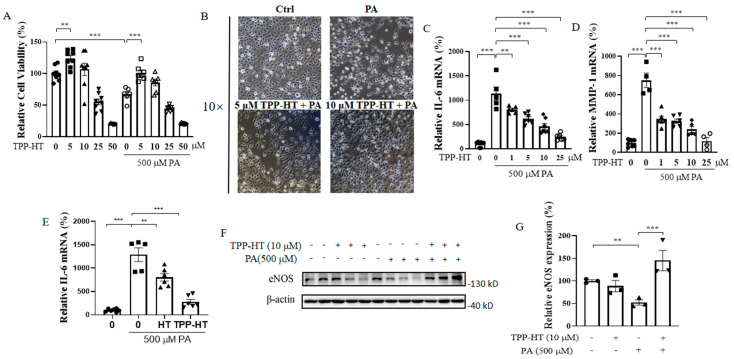
TPP-HT prevents PA-induced cellular lipotoxicity, increases inflammation and decreases eNOS expression in HAECs. HAECs were pretreated with TPP-HT (1, 5, 10, 25, 50 μM), TPP-HT (10 μM) or HT (10 μM) for 24 h, followed by PA (500 μM) challenge for another 24 h. (**A**) Cell viability. (**B**) Bright-field microscope image of cells. (**C**) mRNA level of IL-6. (**D**) mRNA level of MMP-1. (**E**) mRNA level of IL-6. (**F**) Protein levels of eNOS. (**G**) Arbitrary unit for protein levels of eNOS. Values are the mean ± SEM from at least three independent experiments. ** *p* < 0.01, *** *p* < 0.001.

To investigate how TPP-HT ameliorates endothelial damage, our study constructed a cellular model with HAECs and PA. PA challenge was employed to induce lipotoxicity-associated endothelial dysfunction [19]. HAECs were pretreated with TPP-HT (1, 5, 10, 25, 50 μM) for 24 h, followed by PA challenge (500 μM) for another 24 h. The results of CCK-8 detection showed that PA sharply decreased cell viability (67.16 ± 10.42% of the BSA control), and TPP-HT (5 and 10 μM) showed protective effects on cell viability in comparison with the PA-alone-treated group (Figure 2A), which was in accordance with the microscopy results (Figure 2B). Inflammation is the main factor participating in endothelial dysfunction [22]. Inflammatory factors interleukin 6 (IL-6) and matrix metalloproteinase 1 (MMP-1) were drastically increased by PA challenge (1132.0 ± 313.6 and 750.2 ± 150.2% of BSA control, respectively), which were dramatically ameliorated by TPP-HT (5 and 10 μM) treatments (Figure 2C,D). Thus, to reach sufficient protective effects of TPP-HT, we then used 10 μM TPP-HT treatment for 24 h in the following tests. Moreover, the anti-inflammatory effects of TPP-HT were much better than the anti-inflammatory effects of HT in the PA-induced HAEC model (Figure 2E). Normal expression of eNOS in endothelial cells is absolutely essential for the normal functioning of blood vessels [23]. Free fatty acids (FFAs) are associated with endothelial dysfunction, specifically due to abnormalities in the endothelium-derived nitric oxide system [24]. To characterize PA-induced endothelial dysfunction, eNOS expression was detected. As expected, 500 μM PA challenge for 24 h resulted in a sharp decrease in eNOS expression, which was recovered by 10 μM TPP-HT pretreatment (Figure 2F,G), indicating that TPP-HT effectively blocked the impact of PA on eNOS expression.

### 3.3. TPP-HT Upregulates the Mitochondrial ETC Complex II and Prevents PA-Induced ROS Generation, a Drop in ATP Levels and Mitochondrial Dysregulation in HAECs

**Figure 3:**
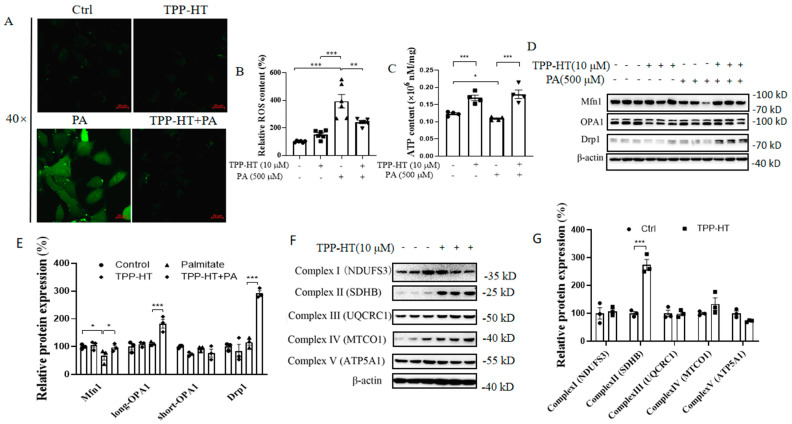
TPP-HT upregulates the mitochondrial ETC complex II and prevents PA-induced ROS generation, a drop in ATP levels and mitochondrial dysregulation in HAECs. HAECs were pretreated or treated with only TPP-HT (10 μM) for 24 h, followed by PA (500 μM) treatment for another 24 h. (**A**) ROS level using a microscope, scale bar = 20 μm. (**B**) ROS level using a microplate fluorometer. (**C**) ATP level. (**D**) Protein levels of mitochondrial membrane fusion- and fission-related proteins. (**E**) Arbitrary unit for protein levels. (**F**) Protein levels of mitochondrial complexes. (**G**) Arbitrary unit for protein levels. Values are the mean ± SEM from at least three independent experiments. * *p* < 0.05, ** *p* < 0.01, *** *p* < 0.001.

The chronic inflammatory response augments ROS production, inducing oxidative stress and further resulting in endothelial dysfunction [25]; therefore, oxidative stress was further detected with the redox-sensitive fluorescent probe DCFH2-DA. Excessive production of ROS was observed in cells exposed to PA (500 μM) for 24 h, and, as expected, pretreatment with TPP-HT (10 μM) significantly abolished PA-stimulated ROS production (Figure 3A,B). From our previous study, the mitochondria-derived ROS is elevated in HAEC cells challenged with PA [19]. The sustained increase in ROS production could be generated from undesirable side products of oxidative energy metabolism, leading to mitochondrial dysfunction, such as a decrease in ATP production [26]. Our results showed that TPP-HT promoted ATP release and rescued PA-induced ATP loss (Figure 3C). Excessive amounts of ROS and decreased ATP levels may arise mainly from less well-regulated mitochondria, such as perturbations of mitochondrial dynamics and the electron-transport chain, which are involved in endothelial damage [27]. Thus, we investigated the effect of TPP-HT on the above mitochondria-related proteins. TPP-HT significantly prevented the PA-induced decrease in the protein level of Mfn1, which is responsible for mitochondrial fusion. Intriguingly, the long-OPA1 and Drp1 were both significantly increased with TPP-HT under PA treatment, whereas PA-alone treatment could not decrease their expression (Figure 3D,E). As shown by our results, the PA-induced decrease in Mfn1 levels were restored by TPP-HT pretreatment, while PA treatment has no influence on the expression of OPA1 and Drp1, and TPP-HT pretreatment could increase the levels of both long-OPA1 and Drp1 in HAEC cells treated with PA, indicating that TPP-HT may promote mitochondrial dynamics. Additionally, for five mitochondrial complexes, TPP-HT pretreatment for 24 h could only efficiently increased expression of mitochondrial complex IIsubunit succinate dehydrogenase (SDHB), a central purveyor of oxidative energy metabolism in mitochondria [28] (Figure 3F,G). Perhaps, TPP-HT could bind to and stabilize SDHB after entering mitochondria. Preliminarily, we evaluated the possibility of the binding of TPP-HT to SDHB, by docking the ligand TPP-HT (stick, in grey) into the proposed substrate-binding pocket of protein SDHB (cartoon, in cyan) and found that TPP-HT shows the best binding pose with the lowest binding energy of −7.6 kcal/mol (Figure S2, indicating that TPP-HT is very likely to interact with SDHB), though further experiments are necessary to verify this.

### 3.4. TPP-HT Prevents PA-Induced Downregulation of FoxO1 and Augments FoxO1 Nuclear Localization in HAECs

**Figure 4:**
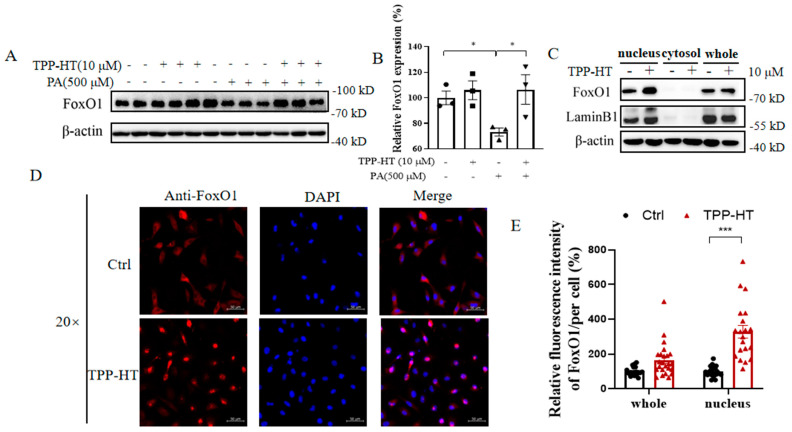
TPP-HT prevents PA-induced downregulation of FoxO1 and augments FoxO1 nuclear localization in HAECs. HAECs were treated with TPP-HT (10 μM) for 24 h and then treated with or without PA (500 μM) for another 24 h. (**A**) Protein level of FoxO1. (**B**) Arbitrary unit for the protein level of FoxO1. (**C**) Protein levels of nuclear, cytosolic and whole levels of FoxO1, LaminB1 as nuclear loading controls. (**D**) Fluorescence intensity of FoxO1, scale bar = 50 μm. (**E**) Arbitrary unit for the fluorescence intensity of FoxO1 (statistical cell number for control is 22, for TPP-HT is 25). Values are the mean ± SEM from at least three independent experiments. * *p* < 0.05, *** *p* < 0.001.

Marked augmentation of ATP production and mitochondrial oxidative respiration by TPP-HT was discovered in our in vitro studies. Moreover, emerging evidence documents FoxO1 as a crucial transcription factor that regulates genes controlling several cellular functions, including mitochondrial function and ATP production [29]. We thereby examined whether FoxO1 was regulated in our model. FoxO1 expression in the presence or absence of TPP-HT was detected, but there were no significant alterations. PA drastically disrupted FoxO1 expression, while the PA-induced disruption was rescued by TPP-HT pretreatment, suggesting that FoxO1 may be involved in TPP-HT cytoprotection (Figure 4A,B). Hence, we further investigated the nuclear translocation of FoxO1. Interestingly, we found that TPP-HT treatment promoted the nuclear translocation of FoxO1, as shown by both subcellular fractionation and immunofluorescence imaging (Figure 4C–E).

### 3.5. FoxO1 Partially Mediates TPP-HT’s Prevention of PA-Induced ATP Drop, ROS Generation and Inflammation in HAECs

**Figure 5:**
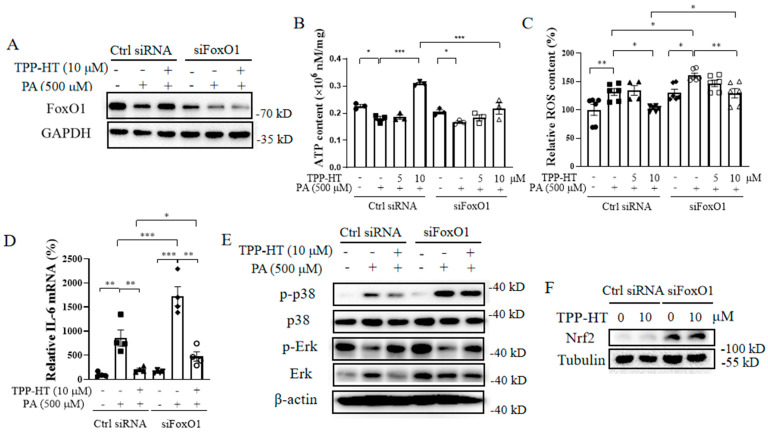
FoxO1 partially mediates the TPP-HT prevention of PA-induced ATP drop, ROS generation and inflammation in HAECs. Ctrl siRNA- or FoxO1 siRNA-transfected HAECs were treated with TPP-HT for 24 h and then treated with or without PA at 500 μM for another 24 h. (**A**) Protein levels of FoxO1. (**B**) ATP content. (**C**) ROS content. (**D**) mRNA level of IL-6. (**E**) Protein levels of p-p38, p38, p-Erk and Erk. (**F**) Protein levels of Nrf2. Values are the mean ± SEM from at least three independent experiments. * *p* < 0.05, ** *p* < 0.01, *** *p* < 0.001.

As shown in the above results, TPP-HT exhibited several protective effects, including inflammation, oxidative stress and energetic dysregulation. To explore whether the cytoprotection of TPP-HT was mainly mediated by the FoxO1 regulator, siRNA sequences were used to knockdown FoxO1 in HAECs. As expected, the upregulated expression of FoxO1 by TPP-HT was abolished by FoxO1-targeting siRNA (Figure 5A). Moreover, 10 μM TPP-HT failed to promote ATP production in HAECs under FoxO1 knockdown conditions (Figure 5B), but siFoxO1 had no influence on the TPP-HT-induced decrease in ROS and IL-6 mRNA levels (Figure 5C,D). Intriguingly, knockdown of FoxO1 exacerbated PA-stimulated ROS release and IL-6 production (Figure 5C,D). These results suggest that TPP-HT-potentiated ATP production is mediated mainly by the activation of the FoxO1 signaling pathway, while the decrease in ROS production by TPP-HT in the current study may involve other signaling pathways. Moreover, FoxO1 knockdown aggravated IL-6 production in all probability by increasing p38 activation. In accordance with the above results, siFoxO1 sharpened p38 activation, suggesting that the exacerbation of PA-induced IL-6 release by siFoxO1 is probably mediated by further activating the p38 pathway (Figure 5E). However, Erk has been reported to be an upstream regulator that directly regulates FoxO1 phosphorylation. The p-Erk/Erk ratio was decreased under siFoxO1 conditions. The transcription factor Nrf2 acts as a pivotal regulator of the cellular antioxidant response [30], while FoxO1 knockdown drastically elevated Nrf2 expression (Figure 5F), indicating that Nrf2 may stimulate the antioxidant network to scavenge excessive ROS production resulting from FoxO1 knockdown.

### 3.6. TPP-HT Pretreatment Abrogates PA-Induced Excessive ROS Production by Promoting Nrf2 Nuclear Localization

**Figure 6:**
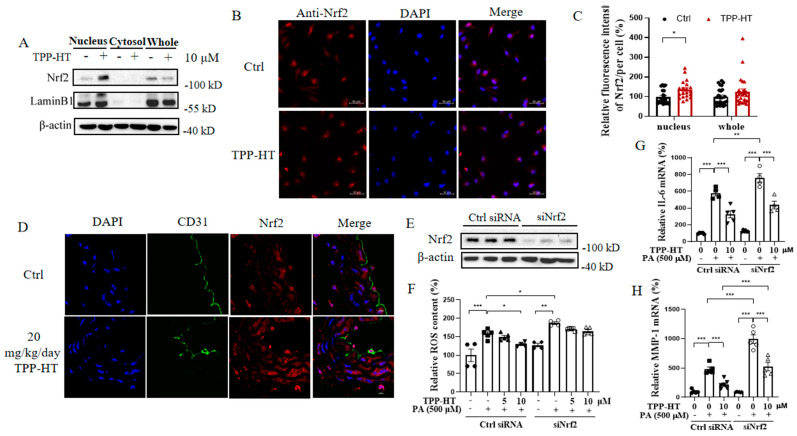
TPP-HT pretreatment abrogates PA-induced excessive ROS production by promoting Nrf2 nuclear localization. (**A**) Protein levels of nuclear, cytosolic and total Nrf2 in HAECs pretreated with TPP-HT (10 μM) for 24 h. (**B**) Fluorescence intensity of FoxO1, scale bar = 50 μm. (**C**) Arbitrary unit for the fluorescence intensity of FoxO1 (statistical cell number for control is 25, for TPP-HT is 25). (**D**) Nrf2 expression by immunofluorescence in the thoracic aorta, CD31 as the biomarker for endothelial cells in vessel, scale bar = 10 μm. Nrf2 expression levels under siNrf2 conditions. (**F**) ROS content. (**G**) mRNA level of IL-6. (H) mRNA level of MMP-1. Values are mean ± SEM from at least three independent experiments. * *p* < 0.05, ** *p* < 0.01, *** *p* < 0.001.

As a master regulator of cellular resistance to oxidative stress, Nrf2 was further considered involved in the antioxidant effects of TPP-HT. The results indicated that TPP-HT had no influence on Nrf2 expression but significantly promoted Nrf2 nuclear translocation (Figure 6A–C), which further induced the expression of an array of antioxidant response element-dependent genes to regulate antioxidant defense [31]. Accordingly, our in vivo findings showed that 20 mg/kg/day TPP-HT for 9 days also promoted Nrf2 nuclear translocation in endothelial cells of the thoracic aorta, suggesting that Nrf2 was a crucial regulator in the presence of TPP-HT (Figure 6D). Moreover, we detected the mRNA levels of Nrf2′s target gene GSTM (glutathione S-transferase mu) by Realtime-q-PCR, and found that TPP-HT could significantly increase mRNA level of GSTM in a dose-dependent manner (Figure S1). Therefore, the knockdown of Nrf2 expression by siRNA was used to elucidate whether it could impede the ability of TPP-HT pretreatment to inhibit the PA-induced ROS burst. As expected, siNrf2 exacerbated the PA-induced inflammatory response; moreover, the inhibition of excessive ROS production by TPP-HT was completely blocked by siNrf2 (Figure 6E,F). However, Nrf2 knockdown did not weaken the anti-inflammatory effects of TPP-HT but aggravated PA-induced IL-6 and MMP-1 production, indicating that the anti-inflammatory effects of TPP-HT in the present study might be regulated via other signaling pathways (Figure 6G,H).

### 3.7. TPP-HT Pretreatment Abrogates PA-Induced IL-6 and MMP-1 mRNA Production by Inhibiting the p38/NF-кB Signaling Pathway in HAECs

**Figure 7:**
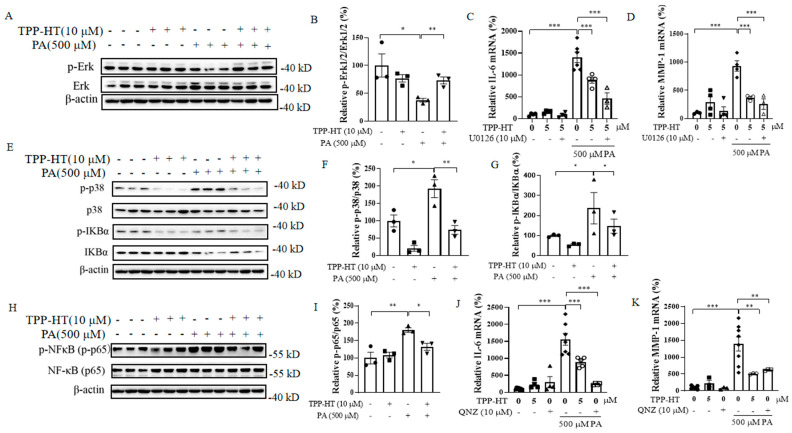
TPP-HT pretreatment abrogates PA-induced IL-6 and MMP-1 mRNA production by inhibiting the p38/NF-кB signaling pathway in HAECs. HAECs were treated with TPP-HT at 10 μM for 24 h, followed by 500 μM PA challenge for 24 h. HAECs were treated with TPP-HT (10 μM) for 24 h in the absence or presence of Erk inhibitor U0126 (10 μM) or NF-кB inhibitor QNZ (10 μM) pretreatment, followed by PA (500 μM) challenge for another 24 h. (**A**) Protein levels of p-Erk, Erk. (**B**) Arbitrary unit for protein levels of p-Erk, Erk. (**C**) mRNA level of IL-6. (**D**) mRNA level of MMP-1. (**E**) Protein levels of p-p38, p38, p-IKBα and IKBα. (**F**) Arbitrary unit for protein levels of p-p38, p38. (**G**) Arbitrary unit for protein levels of p-IKBα and IKBα. (**H**) Protein levels of p-NF-κB and NF-κB. (**I**) Arbitrary unit for protein levels of p-NF-κB and NF-κB. (**J**) mRNA level of IL-6. (**K**) mRNA level of MMP-1. * *p* < 0.05, ** *p* < 0.01, *** *p* < 0.001.

Mitogen-activated protein kinases (MAPKs), which belong to a large family of serine-threonine kinases, play a pivotal role in responding to external stimulation [32]. Extracellular damage activates members of the major MAPK subfamilies, such as Erk and p38. In conjunction with the activation of NF-κB, MAPK activation induces the expression of multiple genes that together regulate the inflammatory response [33]. To further explore the mechanism of the anti-inflammatory effects of TPP-HT, we detected the protein levels of p-Erk, Erk, p-p38 and p38. The results showed that the p-Erk/Erk ratio was sharply decreased by PA challenge, while TPP-HT pretreatment restored the ratio (Figure 7A,B). Furthermore, Erk inhibitor (U0126) pretreatment prior to TPP-HT was used to investigate whether U0126 could eliminate the ability of TPP-HT pretreatment to reduce PA-induced elevation of IL-6 and MMP-1 mRNA levels (Figure 7C,D). Unfortunately, the results showed that Erk pathway activation was not the key regulating signaling pathway for the anti-inflammatory activity of TPP-HT. Generally, a sustained increase in ROS levels results in activation of the p38 pathway [34]. Therefore, changes in the p38 MAPK/NF-κB pathway, which responds sensitively to cellular stimulation and further participates in the inflammatory reaction, were investigated [35]. Additionally, NF-кB, a crucial transcription factor, regulates the initiation of the inflammatory response and controls a broad range of markers, such as IL-6 and MMP-1 [36]. Our results in Figure 7E,F show that PA dramatically promoted the protein level of p-p38, and TPP-HT pretreatment significantly decreased the protein level of p-p38, as well as the p-p38/p38 ratio. Consistently, phosphorylation of IкBα, a downstream target of p38, was sufficiently increased by PA and recovered by TPP-HT pretreatment (Figure 7E,G). The elevated levels of p-IкBα induced by PA may promote the phosphorylation of NF-кB. As expected, the PA-induced dramatic increase in NF-кB phosphorylation was reduced by TPP-HT pretreatment (Figure 7H,I). NF-кB phosphorylation leads to NF-кB nuclear translocation, thus activating downstream inflammatory gene expression. Inhibition of NF-кB activity by QNZ showed comparable effects on inhibiting PA-induced IL-6 and MMP-1 mRNA production in HAECs (Figure 7G,K), which was consistent with previous studies showing that p38 activation could further activate the NF-кB-mediated inflammatory signaling factor IL-6 [37].

## 4. Discussion

Mitochondria have been regarded as one of the most significant targets and hotspots for new drug design, development and utilization in CVDs [38]. To date, one of the most effective methods to deliver drugs specifically to mitochondria is via covalent linking of a lipophilic cation, for instance, linking a triphenylphosphonium moiety to a central pharmacophore [39,40]. Using the above approach, the new drug could penetrate the cell and mitochondrial membranes better; thus, a more than 1000-fold higher mitochondrial concentration could become a reality [41].

In our previous studies, the results showed that 17 weeks of administration of 50 mg/kg HT sufficiently prevented high-fat diet-induced obesity, hyperglycemia and hyperlipidemia and ameliorated mitochondrial dysfunction in C57BL/6 J mice [42]. Diverse authors have demonstrated the cytoprotective effects of HT against inflammation, oxidative stress, etc. [43,44]. In light of this evidence, we de novo designed and synthesized TPP-HT to better exploit the protective effects of the HT structure core on mitochondria. In the current study, we found that TPP-HT has a better anti-inflammatory effect than HT. Moreover, TPP-HT significantly ameliorated hyperlipidemia-induced endothelial and mitochondrial impairment in the thoracic aorta, which is reported for the first time. We tried to elucidate the underlying mechanisms and found that TPP-HT promoted the nuclear translocation of FoxO1 and Nrf2, implying applications of TPP-HT in interventions for other pathologies involving the dysregulation of FoxO1 and Nrf2. This study helps validate and supplement the cardiovascular protection of HT [45,46] and HT derivatives [47,48].

Endothelial FoxO1 plays a key and specific role in vascular homeostasis, and loss of its function cannot be substituted with other FoxO family members [49,50,51]. As a pivotal transcription factor, FoxO1 regulates genes controlling several cellular functions, including mitochondrial function and ATP production [29]. Additionally, FoxO1 plays a significant role during protection against oxidative stress through the induction of endogenous antioxidants; moreover, FoxO1 is a master regulator of inflammatory responses during metabolic disorders, such as obesity and diabetes [52]. Our results identified the potentiation of ATP production via the activation of the FoxO1 signaling pathway as one of the key underlying mechanisms of the protective effects of TPP-HT. Activating the Nrf2 pathway was reported to increase endothelial tolerance to oxidative stress and inflammation, implying that Nrf2 is a key regulator of endothelial function [53]. In the present study, TPP-HT promoted Nrf2 nuclear localization both in vitro and in vivo, and the activation of Nrf2 was the major underlying mechanism of prevention of PA-induced excessive ROS production by TPP-HT, further corroborating that TPP-HT is an efficacious candidate drug for intervention in endothelial dysfunction. From our findings, both FoxO1 and Nrf2 activation underlie the endothelial protective effects of TPP-HT, and we showed that there was a negative regulation between FoxO1 and Nrf2; knocking down FoxO1 upregulates Nrf2 (Figure 5F). Therefore, although FoxO1 and Nrf2 work together to mediate the endothelial protective effects of TPP-HT, other mediators exist that regulate FoxO1 and Nrf2 in response to TPP-HT. In general, among MAPK signaling pathways, Erk activation has been reported to mediate FoxO1 and Nrf2 translocation into nuclei, subsequently inducing downstream energetic and antioxidant gene activity [54,55]. Our data showed that TPP-HT pretreatment prevented the inhibition of phosphorylated Erk1/2, suggesting that Erk1/2 activation may be the upstream regulator of both FoxO1 and Nrf2, which needs to be further studied.

Generally, NF-κB family proteins are important transcription factors that regulate the inflammatory response and are further involved in many physiological and pathological conditions. A variety of cellular processes create ROS as part of cellular signaling events. The crosstalk between ROS and NF-κB signaling is highly context-dependent and has been extensively discussed.

Numerous studies have shown that ROS can lead to the generation of intracellular signals that stimulate the inflammatory response, including the activation of MAPK members, such as Erk and p38 [32]. In conjunction with the activation of NF-κB, MAPK activation induces the expression of multiple genes that regulate the inflammatory response [33]. Our findings indicate that TPP-HT pretreatment abrogates PA-induced IL-6 and MMP-1 mRNA production by inhibiting the p38/NF-кB signaling pathway, while the relationship between the suppression of PA-induced ROS production by TPP-HT and its inhibition of PA-induced p38/NF-кB activation remains to be clarified. Altogether, TPP-HT simultaneously suppresses the activation of p38 MAPK induced by PA and activates FoxO1 and Nrf2, which together combats oxidative stress and inflammation in hyperlipidemia-associated endothelial dysfunction.

Our study left one interesting question open for further studies to investigate: How much does TPP-HT accumulate in mitochondria? In addition, we did not determine whether the nuclear translocation of FoxO1 and Nrf2, in response to TPP-HT, is downstream or upstream of targeting and modulating mitochondria. Meanwhile, CVDs occur both in males and females; we used only male mice in our in vivo study to exclude the effect of estrogen on lipid metabolism, while, in future studies, we will simultaneously use male and female mice to investigate the effect of a potential drug candidate like TPP-HT in hyperlipidemia-induced endothelial dysfunction, so as to reach both wider and more rigorous research conclusions. Moreover, TPP-HT has a biphasic response: protective effects at lower concentrations, and toxic effects at higher concentrations. As a small molecule compound like resveratrol or hydroxytyrosol, which lacks a sole specific target, TPP-HT may interact with multiple or even many bio-macromolecules. Therefore, at lower concentrations, TPP-HT would gather in mitochondria and enhance oxidative phosphorylation and the concomitant generation of ROS; this would trigger signaling network within the cell, and thereby indirectly promotes the expression and nuclear-translocation of FoxO1 and Nrf2, and eventually exert its protective effects; however, at higher concentrations, it might affect the behavior of way too many molecules, including enzymes, receptors or signaling pathways, and hence exhibit a toxic effect. At the current stage, TPP-HT only represent a potential drug candidate, which showed better efficacy compared to hydroxytyrosol (Figure 2E), better efficacy and safety could be achieved by further optimization of the structure.

In brief, our current study provided the first evidence that mitochondria-targeted TPP-HT effectively prevented lipotoxicity-induced endothelial dysfunction by activating FoxO1 and Nrf2 nuclear translocation. In more details, TPP-HT upregulates and activates FoxO1 and Nrf2, and upregulates protein level of ECT Complex II subunit SDHB, thereby enhancing mitochondrial function and redox balance, and attenuates PA-induced inflammatory response by inhibiting the p38/NFκB signaling pathway (Figure S3). Our findings provide novel insight into the drug design strategy for intervention in CVDs.

## Data Availability

The data presented in this study are available in the article.

## Supplementary Materials

The following supporting information can be downloaded at https://www.mdpi.com/article/10.3390/antiox12010175/s1, Table S1: Primers. Figure S1: TPP-HT pretreatment contributes to antioxidant responses via activating Erk signaling pathway. HAECs were treated with or without TPP-HT (1, 5, 10 μM) for 24 h in the presence of Erk inhibitor U0126 (10 μM) pretreatment. mRNA level of GSTM. * *p* < 0.05, *** *p* < 0.001. Figure S2: TPP-HT may target mitochondrial ETC complex II. Analysis of ligand (TPP-HT) binding energy in the protein SDHB (mitochondrial respiratory chain complex II) (PDB code: 7KCM). Figure S3: Proposed model for the protective mechanism of TPP-HT. TPP-HT activates FoxO1 and Nrf2 signaling pathway, also may directly promote Complex II (SDHB) expression, thereby prevents ROS generation, blocks inflammatory response by inhibiting p38/NFκB signaling pathway and eventually attenuates PA-induced endothelial injury.
