# Supplementary material for: Mitochondria-Targeted Triphenylphosphonium-Hydroxytyrosol Prevents Lipotoxicity-Induced Endothelial Injury by Enhancing Mitochondrial Function and Redox Balance via Promoting FoxO1 and Nrf2 Nuclear Translocation and Suppressing Inflammation via Inhibiting p38/NF-кB Pathway
Source: Antioxidants (Basel). 2023 Jan 11;12(1):175. doi: 10.3390/antiox12010175 (PMC9854738; doi:10.3390/antiox12010175)
Supplement: Supplementary file 1 [file antioxidants-12-00175-s001.zip › antioxidants-2088894-supplementary.pdf]

Supplementary Table 1 Primers

Species	Gene	Gene ID	Primer (5'-3')
Human	ACTIN	60	Forward: ATCATGTTTGAGACCTTCAA<br>Reverse: AGATGGGCACAGTGTGGGT
Human	IL-6	3569	Forward: TTTTGTACTCATCTGCACAGC<br>Reverse: GGATTCAATGAGGAGACTTGC
Human	MMP-1	4312	Forward: ACGCCAGATTTGCCAAGAG<br>Reverse: TTGACCCTCAGAGACCTTGGT

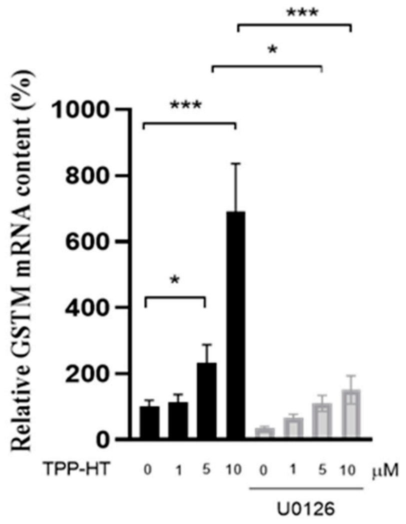

Figure S1 **TPP-HT pretreatment contributes to antioxidant responses via activating Erk signaling pathway.** HAECs were treated with or without TPP-HT (1, 5, 10 μM) for 24 h in the presence of Erk inhibitor U0126 (10 μM) pretreatment. mRNA level of GSTM. *$p < 0.05$, ***$p < 0.001$.

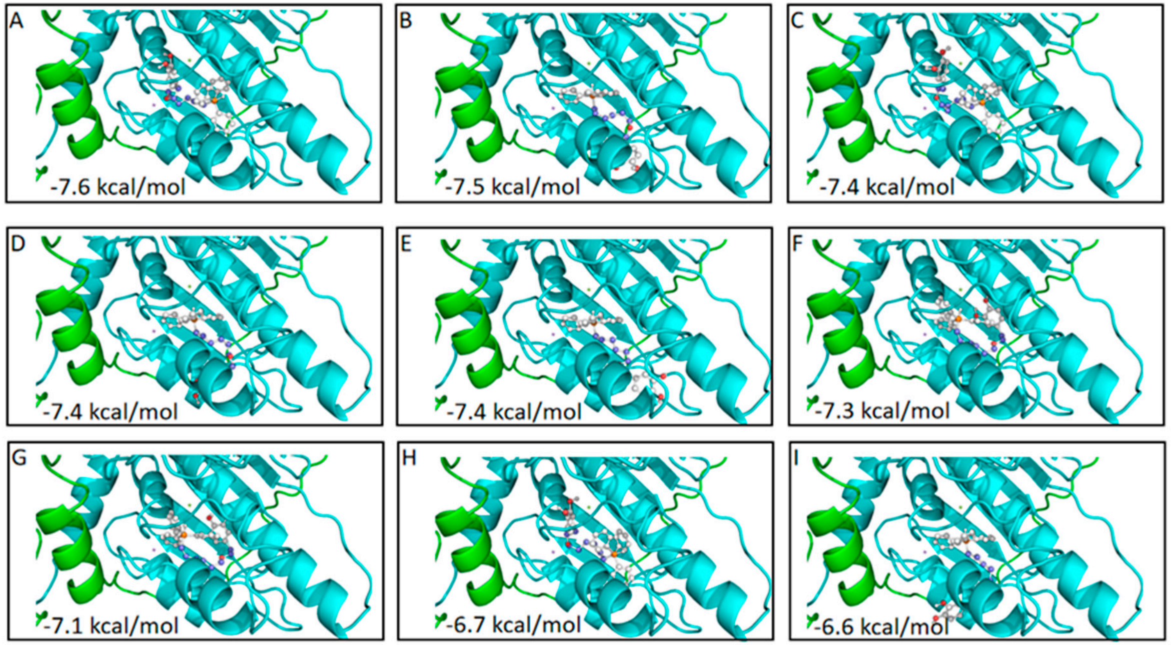

Figure S2 **TPP-HT may target mitochondrial ETC complex II.** Analysis of ligand (TPP-HT) binding energy in the protein SDHB (mitochondrial respiratory chain complex II) (PDB code: 7KCM)

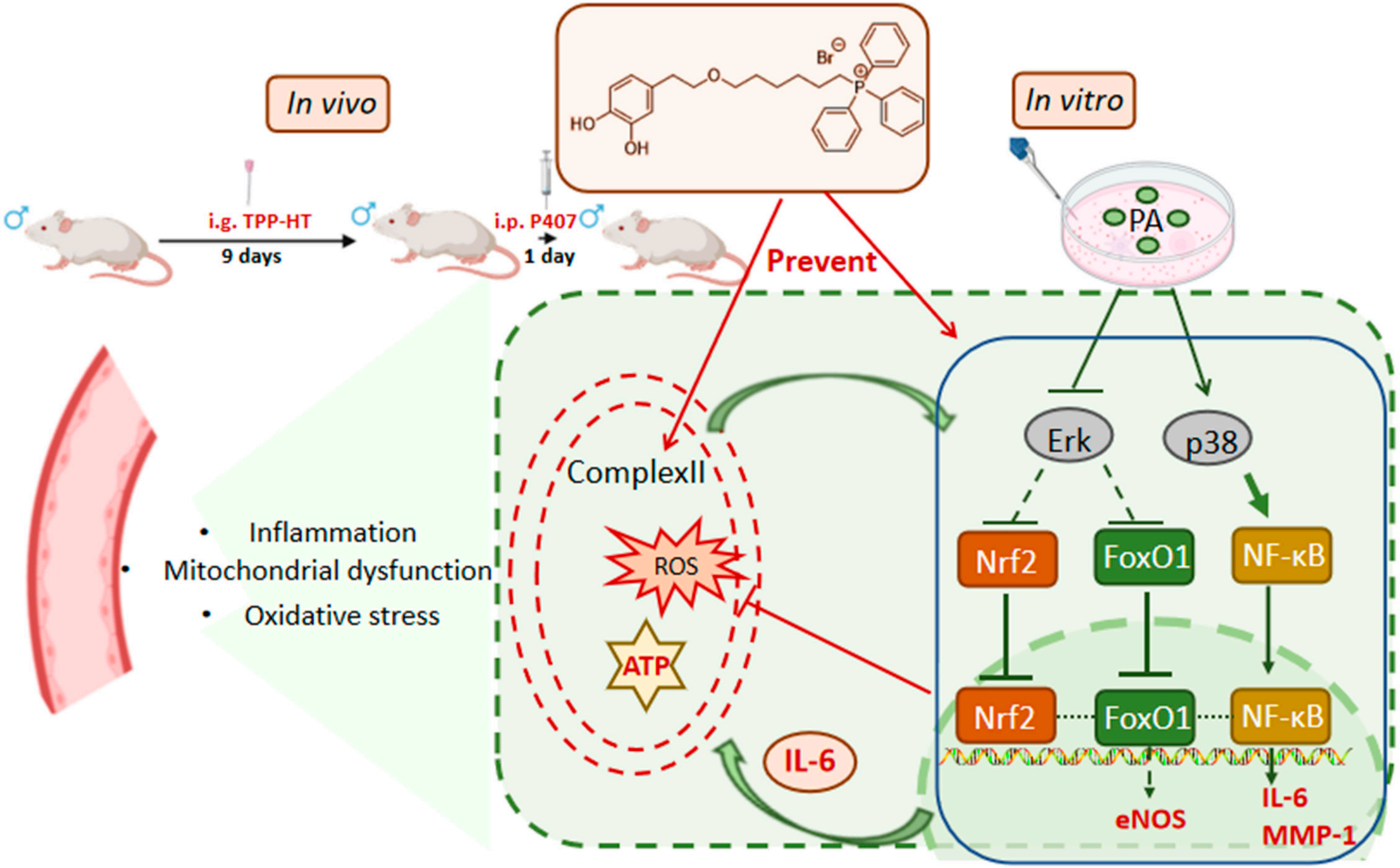

Figure S3 **Proposed model for the protective mechanism of TPP-HT.** TPP-HT activates FoxO1 and Nrf2 signaling pathway, also may directly promote Complex II (SDHB) expression, thereby prevents ROS generation, blocks inflammatory response by inhibiting p38/NFκB signaling pathway and eventually attenuates PA-induced endothelial injury.
